# A Card from the Long Island College Hospital

**Published:** 1870-11

**Authors:** 


### *A Card from the Long Island College Hospital.*

It has recently come to our knowledge that a *quack advertisement* has been been distributed under cover and stamp of the Annual Circular of the Long Island College Hospital.

The Regents and Faculty of the College embrace the earliest opportunity to state that this was done without our knowledge, after the circulars had been committed to the News Agent for distribution.

They deeply regret the necessity for publishing this card; but duty to themselves and the profession requires that this statement be made public.

